# RESULTS FROM A PILOT TRIAL COMPARING MODES OF MEAL DELIVERY TO HOMEBOUND OLDER ADULTS WITH DEMENTIA

**DOI:** 10.1093/geroni/igad104.1637

**Published:** 2023-12-21

**Authors:** Kali Thomas, Jennifer Bunker, Ellen McCreedy, Michelle Hilgeman, Emily Gabois, Roee Gutman

**Affiliations:** Johns Hopkins University, Baltimore, Maryland, United States; Brown University, Providence, Rhode Island, United States; Brown University School of Public Health, Providence, Rhode Island, United States; Tuscaloosa VA Medical Center, Tuscaloosa, Alabama, United States; Brown University School of Public Health, Providence, Rhode Island, United States; Brown University, Providence, Rhode Island, United States

## Abstract

Home-delivered meals promote independence among homebound older adults. However, it is unclear which mode of meal delivery delays nursing home placement among homebound older adults with dementia. We conducted a pilot multisite, two-arm, pragmatic clinical trial in May 2021-May 2022 in which participants with dementia were randomized to receive either: 1) a lunch-time meal delivered 5 times per week or 2) 10 frozen meals that were mailed to participants every two weeks. We used the Minimum Data Set to examine time from randomization to nursing home placement (primary outcome). Our analytic sample included 225 individuals with self-reported dementia on waiting lists at three Meals on Wheels programs in Florida and Texas. 121 participants were randomized to daily-delivered meals and 104 to frozen, drop-shipped meals. At 6 months from randomization, 9(7%) who received daily-delivered meals were admitted to a nursing home, compared to 15(14%) who received frozen, drop-shipped meals p-value=0.14,95%CI(-0.02,0.16). The adjusted log hazard ratio of nursing home placement was -0.65(SE=0.42)p-value=0.12). While this pilot was not adequately powered to detect statistically significant differences between arms, results suggest that even with social distancing requirements and a reduction in the number of deliveries because of the COVID-19 pandemic, there is a trend toward lower nursing home placement among participants with dementia who received daily-delivered meals compared to frozen, drop-shipped meals.

